# Authors’ response to the letter: Takotsubo syndrome: a neurocardiac syndrome inside the autonomic nervous system

**DOI:** 10.1007/s10741-019-09789-y

**Published:** 2019-04-16

**Authors:** Sonia Borodzicz, Katarzyna Czarzasta, Grzegorz Opolski, Agnieszka Cudnoch-Jędrzejewska

**Affiliations:** 1 Department of Experimental and Clinical Physiology, Laboratory of Centre for Preclinical Research, Medical University of Warsaw, 1b Banacha Street, 02-097 Warsaw, Poland; 2 1st Chair and Department of Cardiology, Medical University of Warsaw, Warsaw, Poland

**Keywords:** Takotsubo syndrome, Pathophysiology, Autonomic nervous system, Sympathetic nervous system

We appreciate Dr. Marafioti and Dr. Benfari’s interest and comments concerning our article [1]. Referring to their first raised issue, currently, there is little known about the specific neuronal changes in patients with Takotsubo syndrome (TTS). The neuroimaging studies by Klein et al. performed in patients with a history of TTS revealed structural and functional changes not only in structures involved in modulation of the sympathetic nervous system (supplementary motor area, left paracentral gyrus, left superior parietal lobe, putamen, and hippocampus), but also with the regions regulating activation of the parasympathetic nervous system (right precentral gyrus, precuneus, and medial temporal gyrus) [2]. Moreover, the alterations in brain structures regulating both sympathetic and parasympathetic nervous systems, including the left amygdala, angular gyrus and left insula, were observed [2]. It has been suggested that insula mediated the impairment of the baroreflex control observed in TTS, what may play a pivotal role in the pathogenesis of the disease [2, 3]. Another interesting observation in the context of the role of the insular cortex in autonomic regulation may be a report by Yoshimura et al., in which they showed that in the majority of patients with TTS induced by acute ischemic stroke, the culprit infarcts were localized within the insular cortex [4]. So far, there is no clear data on exactly what type of dysfunction of the autonomic nervous system with the distinction of specific structures underlie TTS, and this issue needs to be further determined.

Discussing the second mentioned point we agree, that anatomical variability of the sympathetic innervation of the heart may be one of the hypotheses explaining various variants of left ventricular wall motion abnormalities in TTS. As we mentioned in our review article, the study performed by Zaroff et al. showed that some of the wall motion patterns in patients with subarachnoid hemorrhage–induced left ventricular systolic dysfunction were related to the distribution of the myocardial sympathetic nerve terminals [1, 5]. Since the exact anatomy of the sympathetic innervation of the heart in different types of TTS has not yet been determined, it seems to be an interesting direction of research aimed at understanding the causes of observed variations.
